# P-1721. Antibiotic Prescribing in Oregon, an Analysis of Outpatient Prescribing Patterns for Acute Respiratory Tract Infections Using Oregon’s All Payer All Claims Database

**DOI:** 10.1093/ofid/ofae631.1885

**Published:** 2025-01-29

**Authors:** Terran Gilbreath, Lisa Iguchi, Elizabeth Breitenstein, Alexia Y Zhang, Paul R Cieslak, Dat Tran

**Affiliations:** Oregon Health Authority - Acute and Communicable Disease Prevention, Portland, Oregon; Oregon Health Authority, Portland, Oregon; Oregon Health Authority, Portland, Oregon; Oregon Health Authority, Portland, Oregon; Oregon Health Authority - Public Health Division, Portland, Oregon; Oregon Health Authority, Portland, Oregon

## Abstract

**Background:**

Acute respiratory tract infections (ARTIs) often lead to antibiotic prescriptions in outpatient settings, many of which are unnecessary. Inappropriate prescribing of antibiotics contributes to the emergence of antibiotic resistance. Antibiotic surveillance related to ARTIs is important for identifying diagnoses and populations for which stewardship efforts are needed. The purpose of this study was to describe antibiotic prescribing trends for ARTIs in outpatient settings in Oregon, both before and during the COVID-19 public health emergency.

**Figure:**
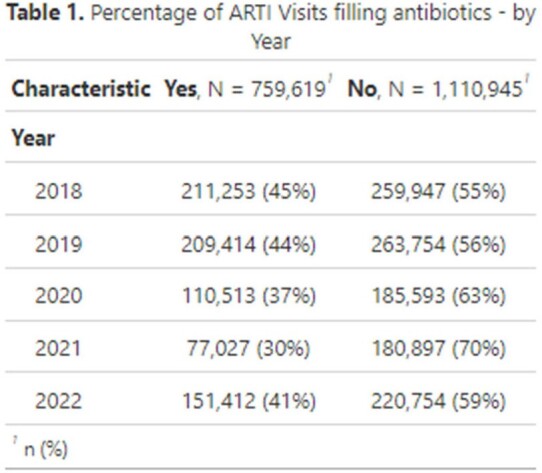

**Methods:**

We queried medical and pharmacy claims during 2018–2022 in Oregon’s All Payer All Claims (APAC) database. Medical claims were restricted to index ARTI visits in outpatient settings. Antibiotic pharmacy claims were matched to ARTI claims using a unique person identifier. We defined “antibiotic use” as ARTI claims that filled an antibiotic prescription within three days of the ARTI visit.

**Figure:**
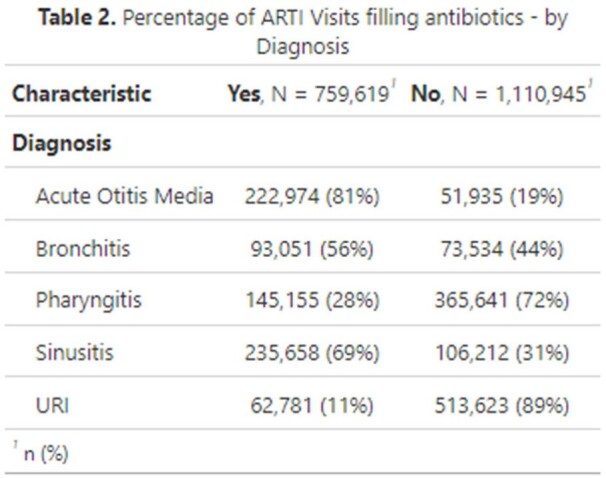

**Results:**

Antibiotic use was observed in 41% of ARTI visits during 2018–2022. Antibiotic use followed approximately 45% of visits in both 2018 and 2019, 37% in 2020, 30% in 2021, and 41% in 2022. Acute otitis media (AOM) had the highest antibiotic use at 81% of visits, followed by sinusitis (69%), bronchitis (56%), pharyngitis (28%), and upper respiratory infections (URI) (11%). Antibiotic use decreased with age for AOM, pharyngitis, and sinusitis - and increased with age for URIs. During 2020-2022, lower antibiotic use for ARTIs was observed, and telehealth use increased. Approximately 27% of ARTI telehealth visits, largely observed in 2020 and 2021, received antibiotics.

**Figure:**
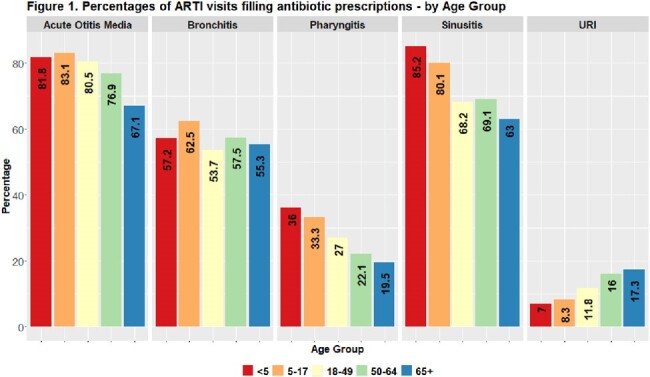

**Conclusion:**

These data are likely confounded by the COVID-19 public health emergency, making comparison to previous analyses difficult. Trends in prescribing for bronchitis, pharyngitis, and URIs showed decreasing antibiotic use from 2018 to 2022. Prescribing trends for sinusitis and bronchitis, where antibiotic use is often unnecessary, indicated these ARTIs as possible targets for stewardship interventions. More broadly, claims data allows public health to monitor prescribing for diagnoses that often do not require antibiotics. Monitoring antibiotic use for ARTIs is important to identify conditions and populations for which stewardship efforts may be needed.

**Figure:**
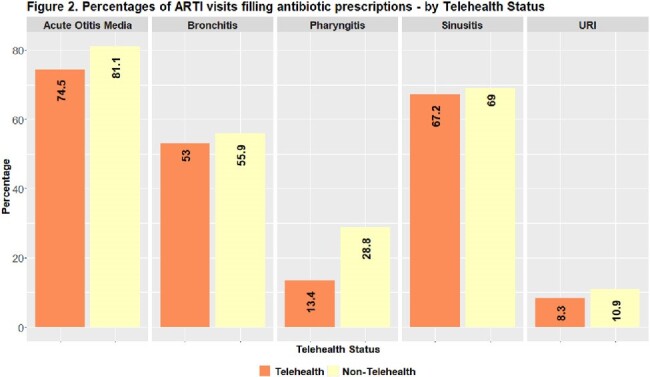

**Disclosures:**

**All Authors**: No reported disclosures

